# Safety and pharmacokinetics of vepdegestrant in Japanese patients with ER+ advanced breast cancer: a phase 1 study

**DOI:** 10.1007/s10147-024-02648-3

**Published:** 2024-11-20

**Authors:** Hiroji Iwata, Yoichi Naito, Masaya Hattori, Akiyo Yoshimura, Kan Yonemori, Mana Aizawa, Yuko Mori, Junichiro Yoshimitsu, Yoshiko Umeyama, Toru Mukohara

**Affiliations:** 1 Department of Breast Oncology, Aichi Cancer Center Hospital, 1-1 Kanokoden, Chikusa-ku, Nagoya, Aichi 464-8681 Japan; 2 Department of Medical Oncology, National Cancer Center Hospital East, 6-5-1 Kashiwanoha, Kashiwa, Chiba 277-8577 Japan; 3 Department of Medical Oncology, National Cancer Center Hospital, 5-1-1 Tsukiji Chuo-ku, Tokyo, 104-0045 Japan; 4 Department of Biometrics and Data Management, Pfizer R&D Japan, 3-22-7 Yoyogi, Shibuya-ku, Tokyo, 151-8589 Japan; 5 Department of Clinical Research, Pfizer R&D Japan, 3-22-7 Yoyogi, Shibuya-ku, Tokyo, 151-8589 Japan; 6 Core Laboratory, Graduate School of Medical Sciences, Department of Medical Research and Developmental Strategy, Nagoya City University, 1 Kawasumi, Mizuho-cho, Mizuho-ku, Nagoya, 467-8601 Japan

**Keywords:** Advanced breast cancer, Estrogen receptor–positive, Human epidermal growth factor receptor 2–negative, Vepdegestrant, Japanese patients, Safety

## Abstract

**Background:**

Vepdegestrant (ARV-471) is an oral PROteolysis TArgeting Chimera (PROTAC) estrogen receptor (ER) degrader.

**Methods:**

This phase 1 study (NCT05463952) investigated safety, pharmacokinetics, and antitumor activity of vepdegestrant in Japanese patients with ER-positive (ER+)/human epidermal growth factor receptor 2–negative (HER2-) advanced breast cancer at the 200-mg once daily (QD) recommended phase 3 dose. Eligible patients had ER+/HER2- advanced breast cancer resistant to standard therapy, with no standard therapy available, or had received two or more prior endocrine therapies in any setting. The primary endpoint was dose-limiting toxicities (DLTs) in cycle 1; secondary endpoints included safety, pharmacokinetics, and antitumor activity.

**Results:**

Six female patients (median age, 58 [range: 47–62] years) were treated. For advanced disease, three (50.0%) patients received three or more prior regimens and five (83.3%) patients received prior cyclin-dependent kinase 4/6 inhibitors. At data cutoff, median treatment duration was 9.8 (range: 6–28) weeks; two patients remained on treatment. No DLTs were observed. Four (66.7%) patients experienced adverse events; none led to dose reduction or discontinuation. Four (66.7%) patients had treatment-related adverse events; all were grade 1 except anemia (grade 2). Geometric mean maximum plasma concentration and 24-h area under the plasma concentration–time curve of vepdegestrant were 630.9 ng/mL and 10,400 ng∙hr/mL after a single dose and 1056 ng/mL and 18,310 ng∙hr/mL after multiple doses. Two (33.3%) patients demonstrated stable disease at week 24.

**Conclusion:**

Vepdegestrant 200 mg QD was well tolerated in Japanese patients with ER+/HER2- advanced breast cancer with no notable differences in pharmacokinetics from Western patients.

**Clinical trial registration:**

ClinicalTrials.gov: NCT05463952 (date of registration: July 19, 2022).

## Introduction

Breast cancer is the most commonly diagnosed cancer worldwide and is a leading cause of cancer death in women [1]. Estrogen receptor–positive (ER+)/human epidermal growth factor receptor 2–negative (HER2-) breast cancer accounts for approximately 65–75% of all breast cancer cases [2, 3], and the expected 5-year survival rate for patients with ER+/HER2- metastatic breast cancer is 34% [4].

The National Clinical Database in Japan collects data on breast cancer from 1423 hospitals, primarily on patients who underwent surgery. In 2018, the database documented 94,999 registered cases of breast cancer in female patients. Among these registered cases, which likely encompasses all Japanese patients with breast cancer who received surgery or initiated treatment in 2018, 53,833 (56.7%) had ER+/HER2- breast cancer (ER and/or HER2 expression data were missing for 24.3% of patients) [5].

The recommended first-line treatment for ER+/HER2- advanced or metastatic breast cancer is endocrine therapy plus a cyclin-dependent kinase (CDK) 4/6 inhibitor [6, 7]. However, many patients with ER+/HER2- breast cancer will eventually develop resistance to current treatments, leading to disease progression and poor patient outcomes [8–12]. Despite resistance to endocrine therapies, the ER pathway remains central to oncogenesis, and is thus a key target for developing new therapeutics to manage ER+/HER2- advanced breast cancer [8, 12].

ER degradation is an approach for treating advanced or metastatic breast cancer [12]. Fulvestrant was the first of a new class of therapies, the selective ER degraders (SERDs), approved for the treatment of ER+/HER2- advanced breast cancer [8]. SERDs indirectly recruit the ubiquitin–proteasome system, secondary to conformational changes and/or immobilization of the ER [12, 13]. However, fulvestrant has several limitations on its use, including intramuscular administration and only 40–50% ER protein degradation at its optimal dose [14–16]. A new oral SERD, elacestrant, has more recently been developed but is currently only approved in the United States and the European Union for use in patients with *ESR1*-mutated ER+/HER2- breast cancer with disease progression following at least one line of endocrine therapy [17, 18].

Vepdegestrant (ARV-471) is a selective, orally administered PROteolysis TArgeting Chimera (PROTAC) ER degrader that directly binds an E3 ubiquitin ligase and ER to trigger proteasomal degradation of ER. Through this ER degradation process, vepdegestrant blocks ER function and downstream signaling and reduces cancer cell proliferation [8, 12]. In contrast to fulvestrant, vepdegestrant treatment yielded substantially greater ER degradation and tumor growth inhibition in a breast cancer xenograft model [19–22].

In the dose escalation, phase 1 portion of a first-in-human phase 1/2 study (NCT04072952), vepdegestrant demonstrated antitumor activity and was well tolerated in heavily pretreated patients with ER+/HER2- advanced breast cancer at all doses tested (30 to 700 mg per day) [23]. In the phase 2 cohort expansion (VERITAC) of this first-in-human study, the selected phase 3 monotherapy dose of vepdegestrant 200 mg once daily (QD) showed durable clinical activity in heavily pretreated patients with ER+/HER2- advanced breast cancer and had a favorable safety profile [24]. The clinical benefit response (defined as complete response, partial response [PR], or stable disease ≥ 24 weeks) was 37.1% (95% confidence interval [CI]: 21.5–55.1%; n = 35). The objective response rate in all evaluable patients (n = 33) was 8.3% (95% CI: 1.0–27.0%); two patients had a confirmed PR. In the vepdegestrant 200 mg cohort (n = 35), 40% of patients received treatment for 24 weeks or longer and 11% for 48 weeks or longer, and no patients required dose reduction due to a treatment-emergent adverse event (TEAE). Most treatment-related adverse events (TRAEs) were grade 1/2, with the most common being fatigue [24].

This phase 1 study (NCT05463952) aimed to investigate the safety, pharmacokinetics, and preliminary efficacy of vepdegestrant in Japanese patients with ER+/HER2- advanced breast cancer at the recommended phase 3 dose (RP3D) of 200 mg QD.

## Material and methods

### Study design

**Fig. 1:**
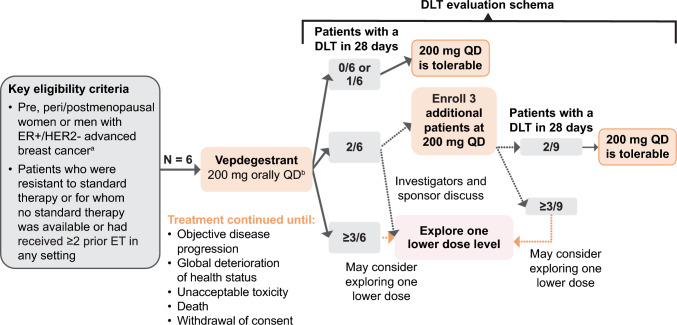
Study design. ^a^Pre- or perimenopausal women or men were eligible to be enrolled if treated with an LHRH agonist for ≥ 4 weeks prior to cycle 1 day 1. If a patient received an LHRH agonist, they were required to remain on it for the duration of the trial. ^b^Vepdegestrant 200 mg was given QD with food in 28-day cycles. *DLT* dose-limiting toxicity, *ER* + estrogen receptor–positive, *ET* endocrine therapy, *HER2-* human epidermal growth factor receptor 2–negative, *LHRH* luteinizing hormone–releasing hormone, *QD* once daily

This was an open-label, nonrandomized, phase 1 study conducted at three sites in Japan. The study used a dose-limiting toxicity (DLT) evaluation scheme (Fig. 1). Initially, six patients were to be enrolled to evaluate DLTs at a dose of vepdegestrant 200 mg QD, which was previously determined to be the RP3D in the first-in-human phase 1/2 study (NCT04072952) [25]. Three additional patients were to be enrolled at the same dose level (200 mg QD) if two of the six patients experienced a DLT. A lower dose level would potentially be explored if more than 33% of patients experienced a DLT at the vepdegestrant 200 mg QD dose.

Vepdegestrant 200 mg QD was taken orally with food. Patients continued the study treatment until objective disease progression, global deterioration of health status, unacceptable toxicity, death, or withdrawal of consent.

### Patients

Eligible patients were aged 20 years or older at the time of screening, were pre-, peri-, or postmenopausal women or men with ER+/HER2- locally advanced or metastatic breast cancer with an Eastern Cooperative Oncology Group performance status of 0 or 1 and adequate bone marrow, coagulation function, and renal/liver function. Patients were required to be resistant to standard therapy, to have no standard therapy available, or to have received two or more prior endocrine therapies in any setting. Pre- or perimenopausal women or men were eligible if treated with a luteinizing hormone–releasing hormone (LHRH) agonist for 4 weeks or more prior to cycle 1 day 1. If a patient had received an LHRH agonist, they were required to remain on it for the duration of the trial. Patients with brain metastases could enroll if they had completed their planned course of treatment, had recovered from acute effects of radiation therapy or surgery, had discontinued high-dose corticosteroid treatment for these metastases for at least 4 weeks, and were neurologically stable per investigator’s judgment. Patients were excluded if they received radiation therapy within 4 weeks of first dose of study drug or had received prior irradiation of more than 25% of the bone marrow. Palliative radiation for the alleviation of pain due to bone metastases was allowed during the study. Patients with any other active malignancy within 3 years prior to enrollment, except for adequately treated basal cell or squamous cell skin cancer, carcinoma in situ of the cervix, or Bowen’s disease, were excluded. Patients were also excluded if they were receiving concurrent administration of medications, foods, or herbal supplements that were strong inhibitors or inducers of cytochrome P450 3A4 (CYP3A4). Prior use was allowed if CYP3A4 inhibitors were stopped at least 7 days prior to enrollment and if strong CYP3A4 inducers were stopped at least 14 days prior to enrollment.

### Study objectives and endpoints

The primary objective was to evaluate the safety and tolerability of vepdegestrant at the RP3D of 200 mg QD. The primary endpoint was DLTs in the first 28-day cycle of treatment. Hematologic DLTs included grade 3 or higher hematologic parameters lasting more than 28 days, grade 3 or higher neutropenia with infection, grade 4 neutropenia lasting more than 5 days, febrile neutropenia, grade 3 thrombocytopenia with bleeding or requiring platelet transfusion, grade 4 thrombocytopenia, or any toxicity requiring dose interruption for 14 days or longer. Nonhematologic DLTs included grade 3 or higher toxicities, Hy’s Law, grade 3 or higher electrolyte abnormality lasting more than 72 h or for any duration if the patient had clinical symptoms, QT corrected by Fridericia’s method prolongation (any grade 3 or higher QT prolongation), any adverse event (AE) attributed to vepdegestrant requiring dose interruption for 14 days or longer, or any death not clearly due to the underlying disease or extraneous causes.

Secondary objectives included evaluating the overall safety profile, characterizing the single-dose and multiple-dose pharmacokinetics of vepdegestrant and ARV-473 (an epimer of vepdegestrant), and exploring preliminary antitumor activity. The safety endpoints were based on incidence of AEs and laboratory abnormalities, characterized by type, frequency, and grade per Common Terminology Criteria for Adverse Events version 5.0 and coded using the Medical Dictionary for Regulatory Activities version 26.0. Pharmacokinetic parameters examined included 24-h area under the plasma concentration–time curve (AUC_24_), maximum observed plasma concentration (C_max_), time to reach maximum concentration (T_max_), predose plasma concentration during multiple dosing (C_trough_), accumulation ratio based on AUC (R_ac_), and effective elimination half-life based on accumulation ratio (t_1/2eff_). The pharmacokinetic endpoints were assessed after single and multiple doses. Samples for pharmacokinetic analysis were obtained before dosing and at 1, 2, 4, 6, 8, 12 (optional), and 24 h post dose on cycle 1 day 1 (after a single dose) and cycle 1 day 15 (after multiple doses). Antitumor activity was evaluated by investigators per Response Evaluation Criteria in Solid Tumors version 1.1 via tumor assessments performed at baseline, every 8 weeks in the first 6 cycles, and every 12 weeks thereafter.

Exploratory objectives included characterization of baseline *ESR1* mutational status and assessment of pharmacodynamic effects on exploratory biomarkers. The exploratory endpoints included *ESR1* mutational status in circulating tumor DNA (ctDNA) and/or tumor tissue. Plasma samples for ctDNA analysis were collected on day 1 of cycles 1, 3, and 6, and at the end of treatment. Sequencing was performed with the FoundationOne Liquid CDx test.

### Statistical analysis

All patients who received at least 75% of the planned dose intensity of study treatment or experienced a DLT during the DLT observation period (first 28-day cycle of treatment) were included in the DLT evaluable set. All patients who received at least one dose of study treatment were included in the safety analysis set. All patients who received at least one dose of the study treatment and had adequate baseline disease assessment were included in the response evaluation set. All patients who were treated and had at least one analyte concentration were included in the pharmacokinetic analysis set.

Data were summarized descriptively. Pharmacokinetic parameters were derived from concentration–time profiles using noncompartmental analysis methods, as data permitted. There were no statistical hypotheses in this study, therefore no formal statistical testing was performed.

## Results

### Patients

Six female patients (median age, 58 years [range: 47–62]) with ER+/HER2- advanced breast cancer were enrolled from August to November 2022. Patient baseline characteristics are shown in Table 1. The median number of prior regimens in the advanced/metastatic setting was 2.5 (range: 1–10). Prior therapies included treatment with a CDK4/6 inhibitor (5 [83.3%] patients), aromatase inhibitor (5 [83.3%] patients), fulvestrant (4 [66.7%] patients), and chemotherapy (2 [33.3%] patients).

**Table 1:** Baseline characteristics

Characteristic	Total (N = 6)
Sex, n (%)	
Female	6 (100)
Median age (range), years	58 (47–62)
Median weight (range), kg	53.2 (41.9–70.0)
ECOG PS, n (%)	
0	4 (66.7)
1	2 (33.3)
TNM at initial diagnosis, n (%)	
I	1 (16.7)
II	2 (33.3)
III	1 (16.7)
IV	1 (16.7)
Unknown	1 (16.7)
Target disease at baseline, n (%)	
Liver	4 (66.7)
Breast	1 (16.7)
Lung	1 (16.7)
Lymph node	1 (16.7)
None	1 (16.7)
Nontarget disease at baseline, n (%)	
Lymph node	4 (66.7)
Bone	3 (50.0)
Lung	3 (50.0)
Liver	2 (33.3)
Brain	1 (16.7)
Pleura	1 (16.7)
Baseline *ESR1* mutation, n (%)	
Yes	2 (33.3)
No	3 (50.0)
Unknown	1 (16.7)
Number of prior regimens, median (range)	
Any setting	4.5 (2–14)
Advanced/metastatic setting	2.5 (1–10)
Lines of prior regimens for advanced/metastatic disease, n (%)	
1	1 (16.7)
2	2 (33.3)
≥ 3	3 (50.0)
Type of prior therapy for advanced/metastatic disease, n (%)	
CDK4/6 inhibitor	5 (83.3)
Aromatase inhibitor	5 (83.3)
Fulvestrant	4 (66.7)
Chemotherapy	2 (33.3)

*CDK* cyclin-dependent kinase, *ECOG PS* Eastern Cooperative Oncology Group performance status, *ESR1* estrogen receptor 1 gene, *TNM* tumor, node, and metastasis

As of the data cutoff of May 4, 2023, all patients started at least two cycles of treatment (median treatment duration, 9.8 weeks [range: 6–28]). Two (33.3%) patients received six or more cycles of treatment and were still on treatment; four (66.7%) patients had discontinued treatment due to progressive disease. In cycle 1, the median relative dose intensity was 100% (range: 100–100%), and across all cycles, median relative dose intensity was 100% (range: 88.9–102.4%).

### Safety

No DLTs in cycle 1 (primary endpoint) were observed in any of the six patients. Overall, four (66.7%) patients experienced TEAEs. All events were reported as grade 1 or 2. There were no serious AEs, and no TEAEs were associated with permanent discontinuations or dose reductions. One patient had a temporary dose interruption of 22 days due to a TEAE (COVID-19); it was not considered treatment related. TRAEs were observed in four (66.7%) patients (Table 2). All reported TRAEs were grade 1, with the exception of one patient with grade 2 anemia. No patients experienced clinically significant QT prolongation.

**Table 2:** Treatment-related adverse events (all cycles)

	Vepdegestrant 200 mg QD (N = 6)			
n (%)	All grades	Grade 1	Grade 2	Grade 3/4
Any treatment-related adverse event	4 (66.7)	3 (50.0)	1 (16.7)	0
Abdominal discomfort	1 (16.7)	1 (16.7)	0	0
Anemia	1 (16.7)	0	1 (16.7)	0
Dizziness	1 (16.7)	1 (16.7)	0	0
Increased ALT	1 (16.7)	1 (16.7)	0	0
Increased AST	1 (16.7)	1 (16.7)	0	0
Nausea	1 (16.7)	1 (16.7)	0	0
Pruritus	1 (16.7)	1 (16.7)	0	0

Patients may have experienced more than one treatment-related adverse event

*ALT* alanine aminotransferase, *AST* aspartate aminotransferase, *QD* once daily

### Pharmacokinetics

**Fig. 2:**
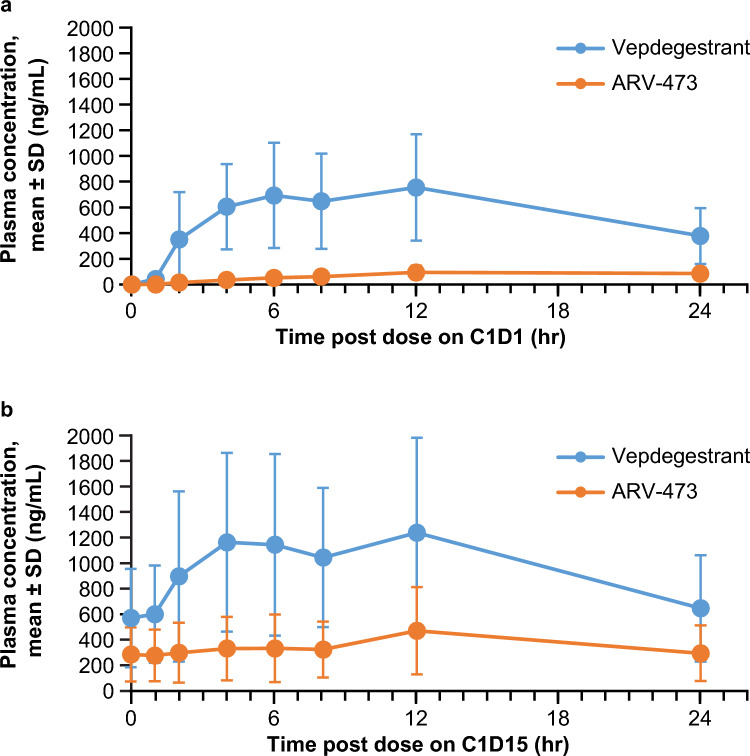
Vepdegestrant and ARV-473 plasma concentration profiles following (**a**) a single dose and (**b**) multiple once-daily doses (N = 6). *C* cycle, *D* day, *SD* standard deviation

The pharmacokinetic profiles of vepdegestrant and ARV-473 were assessed in samples from all patients in the study. The mean plasma concentration profiles of vepdegestrant and ARV-473 after a single dose and multiple doses are shown in Fig. 2. Vepdegestrant median T_max_ was similar after a single dose (cycle 1 day 1) and after multiple QD doses (cycle 1 day 15) in Japanese patients: 4.74 and 4.69 h, respectively. The geometric mean C_max_ and AUC_24_ of vepdegestrant after a single dose were 630.9 ng/mL and 10,400 ng∙hr/mL, respectively. After multiple QD doses, geometric mean C_max_ and AUC_24_ of vepdegestrant was 1056 ng/mL and 18,310 ng∙hr/mL, respectively. The resulting R_ac_ was 1.76, and the corresponding t_1/2eff_ was 20.2 h (Table 3). Vepdegestrant appeared to reach steady state by cycle 1 day 8, consistent with the t_1/2eff_ value.

**Table 3:** Vepdegestrant and ARV-473 pharmacokinetic parameters following a single dose and multiple once-daily doses

	Vepdegestrant 200 mg QD (N = 6)			
Analyte	Vepdegestrant		ARV-473^a^	
Parameters (unit)	Single dose	Multiple doses	Single dose	Multiple doses
C_max_ (ng/mL)	630.9 (57)	1056 (54)	74.08 (61)	292.5 (69)
C_trough_ (ng/mL)	NA	496.3 (57)	NA	238.1 (69)
T_max_ (hr)	4.74 (3.75–6.28)	4.69 (3.72–6.03)	23.5 (23.0–24.1)	6.83 (3.83–12.0)
t_1/2eff_ (hr)	NA	20.23 ± 6.70	NA	71.60 ± 10.84
AUC_24_ (ng•hr/mL)	10,400 (58)	18,310 (57)	1289 (61)	6175 (70)
R_ac_	NA	1.760 (21)	NA	4.790 (13)

All data are shown as the geometric mean (geometric % coefficient of variation), except T_max_, which is shown as median (range), and t_1/2eff_, which is shown as arithmetic mean ± standard deviation

*AUC*_*24*_ 24-h area under the plasma concentration–time curve, *C*_*max*_ maximum observed plasma concentration, *C*_*trough*_ predose plasma concentration during multiple dosing, *NA* not applicable, *QD* once daily, *R*_*a*c_ ratio of AUC_24_ after multiple doses/AUC_24_ after a single dose, *t*_*1/2eff*_ effective elimination half-life based on accumulation ratio, *T*_*max*_ time to reach C_max_

^a^ARV-473 is an epimer of vepdegestrant

For ARV-473, an epimer of vepdegestrant, median T_max_ after a single dose (cycle 1 day 1) and after multiple QD doses (cycle 1 day 15) was 23.5 and 6.83 h, respectively. The geometric mean C_max_ and AUC_24_ after a single dose were 74.1 ng/mL and 1289 ng∙hr/mL, respectively. After multiple QD doses, geometric mean C_max_ and AUC_24_ of ARV-473 was 292.5 ng/mL and 6175 ng∙hr/mL, respectively. The resulting R_ac_ was 4.79, and the corresponding t_1/2eff_ was 71.6 h (Table 3). The metabolite-to-parent ratios for C_max_ and AUC_24_ were 0.2770 and 0.3374, respectively.

One patient showed higher exposure of vepdegestrant than other patients, with C_max_ of 2490 ng/mL after multiple doses; however, this was not associated with the frequency or severity of AEs. In this patient, only grade 1 dizziness was reported as a TRAE.

### Antitumor activity

**Fig. 3:**
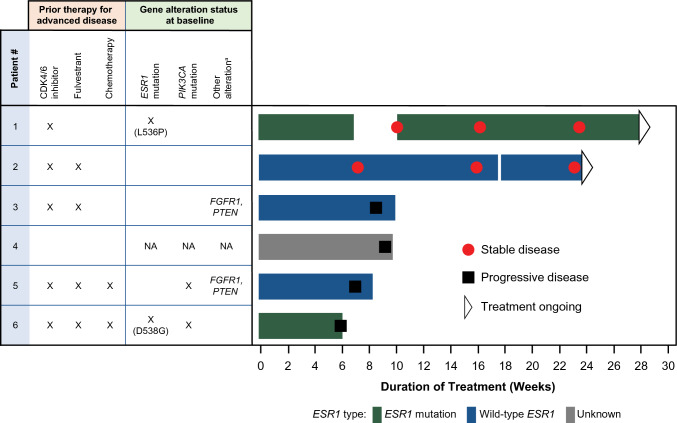
Investigator-assessed tumor response. For patient 4, insufficient amounts of DNA were available for ctDNA analysis. ^a^Not a full list of all genetic alterations detected; genes shown are those associated with endocrine resistance when mutated and/or amplified [12]. *CDK* cyclin-dependent kinase, *ctDNA* circulating tumor DNA, *ESR1* estrogen receptor 1 gene, *FGFR1* fibroblast growth factor receptor 1 gene, *NA* not available, *PIK3CA* phosphatidylinositol-4,5-bisphosphate 3-kinase catalytic subunit alpha gene, *PTEN* phosphatase and tensin homolog gene

All patients were evaluable for tumor response. Two (33.3%) patients demonstrated stable disease at the week 24 tumor assessment. Four (66.7%) patients had progressive disease (Fig. 3). Treatment was ongoing in the two patients with stable disease at the time of data cutoff.

### Biomarker analyses

**Fig. 4:**
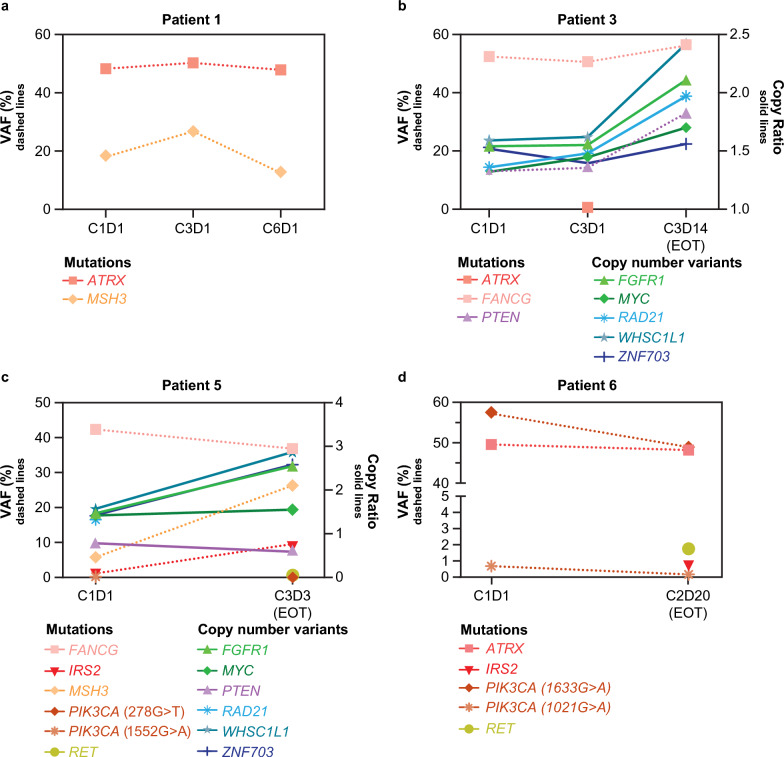
Alterations in key non-*ESR1* genes in individual patients**.** Key genes include *ATRX*, *FANCG*, *FGFR1*, *IRS2*, *MSH3, MYC*, *PIK3CA*, *PTEN*, *RAD21*, *RET*, *WHSC1L1*, and *ZNF703*. No alterations in these key genes were observed for patient 2. For patient 4, insufficient amounts of DNA were available for ctDNA analysis. Patient 1 was on treatment as of the cutoff date, thus no EOT sample was available. *ATRX* ATRX chromatin remodeler gene, *C* cycle, *D* day, *ctDNA* circulating tumor DNA, *EOT* end of treatment, *ESR1* estrogen receptor 1 gene, *FANCG* FA complementation group G gene, *FGFR1* fibroblast growth factor receptor 1 gene, *IRS2* insulin receptor substrate 2 gene, *MSH3* mutS homolog 3 gene, *MYC* MYC proto oncogene bHLH transcription factor gene, *PIK3CA* phosphatidylinositol-4,5-bisphosphate 3-kinase catalytic subunit alpha gene, *PTEN* phosphatase and tensin homolog gene, *RAD21* RAD21 cohesin complex component gene, *RET* ret proto oncogene, *VAF* variant allele fraction, *WHSC1L1* nuclear receptor binding SET domain protein 3 gene, *ZNF703* zinc finger protein 703 gene

**Fig. 5:**
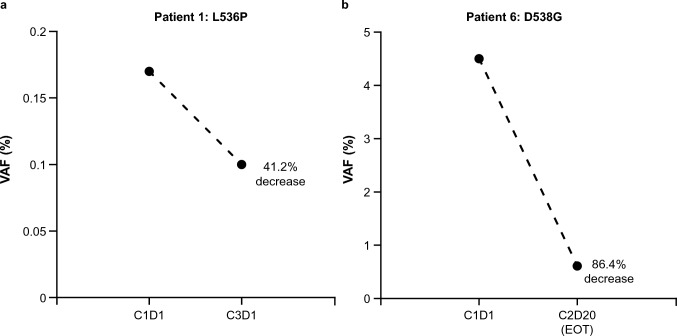
Change in *ESR1* variant allele fraction in individual patients**.** Plots show the change in *ESR1* VAF in 2 patients. Patient 1 was on treatment as of the cutoff date, thus no EOT sample was available. *C* cycle, *D* day, *EOT* end of treatment, *ESR1* estrogen receptor 1 gene, *VAF* variant allele fraction

Five patients were evaluated for one or more of the biomarkers at pre- and/or post dose. The profiles of gene mutations and copy number variations in key non-*ESR1* genes of interest in individual patients across the study are shown in Fig. 4. Genes with alteration(s) on cycle 1 day 1 and/or post dosing in two or more patients were selected for analysis. At baseline, two patients had *ESR1* mutation-positive ctDNA (Fig. 5). For patient 1 (L536P mutation), variant allele fraction (VAF) decreased from 0.17 at baseline to 0.10 at cycle 3, day 1 (41.2% decrease) (Fig. 5a). For patient 6 (D538G mutation), VAF was reduced from 4.50 at baseline to 0.61 at the end of treatment (86.4% decrease) (Fig. 5b).

## Discussion

In this phase 1 study, six female Japanese patients were treated with vepdegestrant at the RP3D of 200 mg QD. This dose was well tolerated in Japanese patients with ER+/HER2- advanced breast cancer and is considered the recommended dose for Japanese patients. No new safety signals with vepdegestrant were seen in Japanese patients and no DLTs were observed. All TEAEs were grade 1 or grade 2 and there were no dose reductions or discontinuations.

Overall, the enrolled patients were heavily pretreated; three (50.0%) patients had received three or more prior regimens for advanced disease, and five (83.3%) patients received prior CDK4/6 inhibitors for advanced disease. Treatment of patients with heavily pretreated, advanced disease is complex [6, 7, 10]. Thus, interpretation of the preliminary antitumor activity reported here is limited by factors such as the small sample size, the heterogeneous characteristics of the patients (eg, prior therapies, gene alteration status at baseline), and the short duration of follow-up. However, it was notable that two patients demonstrated stable disease at the week 24 tumor assessment and remained on treatment at the time of data cutoff.

There were no obvious differences in the pharmacokinetic profiles observed in Japanese patients and those observed in Western patients [23]. In Western patients who received vepdegestrant 200 mg QD (n = 8) in the dose escalation cohort of the first-in-human phase 1/2 study (NCT04072952), geometric mean C_max_ and AUC_24_ were 866 ng/mL and 15,459 ng∙hr/mL, respectively, on day 15. In that study, dose-dependent increases were seen for vepdegestrant exposure across total daily doses from 30 mg up to 500 mg.

In the exploratory biomarker analysis of this study, two patients, one with an L536P mutation and one with a D538G mutation, had reduced *ESR1* VAF after treatment with vepdegestrant. This is consistent with substantial on-treatment reductions in mutant *ESR1* ctDNA levels observed after 1 cycle and sustained over multiple cycles in the dose escalation, phase 1 portion of the first-in-human phase 1/2 study of vepdegestrant (NCT04072952) [23]. In preclinical studies, vepdegestrant induced degradation of multiple clinically relevant ER mutants including Y537S, D538G, Y537C, Y537N, E380Q, L536P, and V422del [22]; however, the relevance of this degradation in the clinical setting has not yet been determined. Furthermore, in the current study, the profiles of some gene alterations, including those in *FGFR1*, *PIK3CA*, *PTEN,* and *RET* changed during the study treatment. Alterations in these genes are of particular interest due to their potential to act as mechanisms of resistance to ER-targeted therapies [12, 26]. Further evaluation of these biomarkers in a larger population is warranted.

In conclusion, this study showed that vepdegestrant at the RP3D of 200 mg QD was well tolerated in Japanese patients with ER+/HER2- advanced breast cancer. Based on this finding, enrollment of Japanese patients is currently ongoing in two global, randomized phase 3 studies of vepdegestrant in patients with ER+/HER2- advanced breast cancer, with sites in the Asia–Pacific region. The VERITAC-2 study (NCT05654623) is comparing the efficacy and safety of vepdegestrant versus fulvestrant in patients with prior CDK4/6 inhibitor therapy and endocrine therapy. The VERITAC-3 study (NCT05909397) will evaluate first-line vepdegestrant with palbociclib versus letrozole with palbociclib in patients with ER+/HER2- advanced breast cancer; a study lead-in assessing two doses of palbociclib (100 mg or 75 mg) with vepdegestrant is ongoing.

## Data Availability

Raw data for this study were generated at Pfizer R&D Japan. Upon request, and subject to review, Pfizer will provide the data that support the findings of this study. Subject to certain criteria, conditions, and exceptions, Pfizer may also provide access to the related individual, de-identified participant data. See https://www.pfizer.com/science/clinical-trials/trial-data-and-results for more information.
